# Anti–Tumor Necrosis Factor Therapy and Risk of Kidney Function Decline and Mortality in Inflammatory Bowel Disease

**DOI:** 10.1001/jamanetworkopen.2024.6822

**Published:** 2024-04-16

**Authors:** Keiichi Sumida, Prabin Shrestha, Yamini Mallisetty, Fridtjof Thomas, Geeta Gyamlani, Elani Streja, Kamyar Kalantar-Zadeh, Csaba P. Kovesdy

**Affiliations:** 1 Division of Nephrology, Department of Medicine, University of Tennessee Health Science Center, Memphis; 2 Division of Biostatistics, Department of Preventive Medicine, College of Medicine, University of Tennessee Health Science Center, Memphis; 3 Nephrology Section, Memphis VA Medical Center, Memphis, Tennessee; 4 Division of Nephrology, Hypertension, and Kidney Transplantation, Department of Medicine, University of California Irvine School of Medicine, Orange; 5 Tibor Rubin Veterans Affairs Medical Center, Long Beach, California

## Abstract

**Importance:**

Inflammatory bowel disease (IBD) is associated with adverse clinical outcomes, including chronic kidney disease and mortality, due in part to chronic inflammation. Little is known about the effects of anti–tumor necrosis factor (TNF) therapy on kidney disease progression and mortality among patients with new-onset IBD.

**Objective:**

To examine the association of incident use of TNF inhibitors with subsequent decline in kidney function and risk of all-cause mortality.

**Design, Setting, and Participants:**

This retrospective cohort study used data from the US Department of Veterans Affairs health care system. Participants were US veterans with new-onset IBD enrolled from October 1, 2004, through September 30, 2019. Data were analyzed from December 2022 to February 2024.

**Exposures:**

Incident use of TNF inhibitors.

**Main Outcomes and Measures:**

The main outcomes were at least 30% decline in estimated glomerular filtration rate (eGFR) and all-cause mortality.

**Results:**

Among 10 689 patients (mean [SD] age, 67.4 [12.3] years; 9999 [93.5%] male) with incident IBD, 3353 (31.4%) had diabetes, the mean (SD) baseline eGFR was 77.2 (19.2) mL/min/1.73 m^2^, and 1515 (14.2%) were newly initiated on anti-TNF therapy. During a median (IQR) follow-up of 4.1 (1.9-7.0) years, 3367 patients experienced at least 30% decline in eGFR, and over a median (IQR) follow-up of 5.0 (2.5-8.0) years, 2502 patients died. After multivariable adjustments, incident use (vs nonuse) of TNF inhibitors was significantly associated with higher risk of decline in eGFR (adjusted hazard ratio [HR], 1.34 [95% CI, 1.18-1.52]) but was not associated with risk of all-cause mortality (adjusted HR, 1.02 [95% CI, 0.86-1.21]). Similar results were observed in sensitivity analyses.

**Conclusions and Relevance:**

In this cohort study of US veterans with incident IBD, incident use (vs nonuse) of TNF inhibitors was independently associated with higher risk of progressive eGFR decline but was not associated with risk of all-cause mortality. Further studies are needed to elucidate potentially distinct pathophysiologic contributions of TNF inhibitor use to kidney and nonkidney outcomes in patients with IBD.

## Key Points

**Question:**

Is anti–tumor necrosis factor (TNF) therapy associated with kidney disease progression or mortality among patients with new-onset inflammatory bowel disease (IBD)?

**Findings:**

In this cohort study of 10 689 US veterans with new-onset IBD, incident use of TNF inhibitors was independently associated with higher risk of progressive kidney function decline but was not associated with all-cause mortality.

**Meaning:**

These findings suggest potentially distinct pathophysiologic contributions of TNF inhibitor use associated with kidney outcomes in patients with IBD and that there is a need for careful monitoring of kidney function when initiating anti-TNF therapy in patients with incident IBD.

## Teaser

This cohort study examines the association of incident use of tumor necrosis factor (TNF) inhibitors with subsequent decline in kidney function and risk of all-cause mortality among patients with new-onset inflammatory bowel disease (IBD).

## Introduction

Inflammatory bowel disease (IBD), including Crohn disease and ulcerative colitis, is a chronic inflammatory disorder of the gastrointestinal tract.^1^ This condition has been associated with poor clinical outcomes, including reduced quality of life, chronic kidney disease (CKD) progression, and premature mortality.^2,3,4,5^ The introduction of biologic agents, such as anti–tumor necrosis factor (TNF) agents, has revolutionized the management of IBD by demonstrating their good efficacy and tolerance, along with various therapeutic benefits, including rapid clinical response and steroid-sparing effects.^6,7,8,9,10^ These benefits have in turn led to the widespread use of biologic agents in the treatment of IBD; however, concerns still exist about its long-term safety, including the risk of a number of autoimmune-mediated phenomena with or without kidney involvement.^11,12,13^

To date, a number of studies have reported favorable effects of anti-TNF therapy on the extraintestinal manifestations of IBD.^14,15,16^ Despite this, studies investigating the effect of anti-TNF therapy on survival for patients with IBD are limited, and to our knowledge, no prior studies have assessed its effect on the risk of CKD progression, a well-known extraintestinal manifestation of IBD and an established risk factor for mortality,^17,18^ in these patients. A few studies investigating the effects of biologic therapy on kidney function in non-IBD populations have reported mixed findings.^19,20,21^ Existing studies on the association of anti-TNF therapy with mortality in IBD have yielded mixed evidence, with a few studies suggesting its survival benefits,^22,23^ while a few others did not find statistically significant associations.^10,24,25^ These mixed findings may be explained at least in part by heterogeneities of study designs and populations across different studies, such as the ascertainment of incident vs prevalent TNF inhibitor use, comparison of TNF inhibitors with vs without active comparators (eg, corticosteroids), and enrollment of patients with incident vs prevalent IBD.

Therefore, to determine the associations of TNF inhibitor use with kidney and survival outcomes in IBD, we used a large nationwide cohort of US veterans with comprehensive patient-level clinical data and investigated the association of incident or new anti-TNF therapy with subsequent risk of kidney disease progression and all-cause mortality in patients with incident IBD, while mitigating confounding by medical indication.

## Methods

This cohort study was approved by the institutional review boards of the Memphis and Long Beach Department of Veterans Affairs (VA) Medical Centers, with exemption from informed consent because there was minimal risk for cohort participants. This report follows the Strengthening the Reporting of Observational Studies in Epidemiology (STROBE).

### Cohort Definition and Study Design

**Figure 1.**
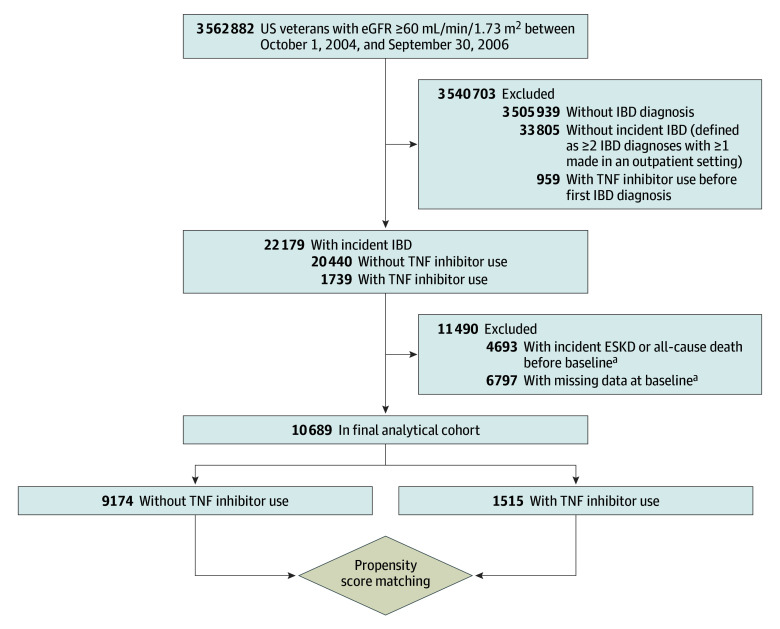
Study Cohort Enrollment Flowchart eGFR indicates estimated glomerular filtration rate; ESKD, end-stage kidney disease; and IBD, inflammatory bowel disease. ^a^Baseline date was the first date of tumor necrosis factor (TNF) inhibitor prescription (for users), or a randomly assigned data that was computer-generated based on the start dates in the treated group (for nonusers) to mitigate differential start of follow-up bias.

We analyzed longitudinal data from the Therapeutic Interventions to Assess Outcomes and Disparities in CKD study, a nationwide retrospective cohort study of 3 562 882 US veterans with estimated glomerular filtration rate (eGFR) 60 mL/min/1.73 m^2^ or greater recorded between October 1, 2004, and September 30, 2006, with follow-up through September 30, 2019.^26^ Since we used an incident new user design to define treatment exposure among patients with incident IBD diagnosis, we first identified 22 179 patients with a new diagnosis of IBD (defined stringently as ≥2 IBD diagnoses, based on *International Classification of Diseases, Ninth Revision* [*ICD-9*] or *International Statistical Classification of Diseases and Related Health Problems, Tenth Revision *[*ICD-10*], after cohort entry with ≥1 diagnosis being made at an outpatient setting^23^ and no evidence of IBD diagnosis prior to cohort entry) (eTable 1 in Supplement 1) and had no documented dispensations of TNF inhibitors from a VA pharmacy anytime before the first IBD diagnosis (Figure 1). After excluding 4693 patients who developed end-stage kidney disease (ESKD) (ie, initiation of dialysis or pre-emptive kidney transplant identified from the US Renal Data System^27^) or died prior to baseline (ie, the first date of de novo TNF inhibitor prescription or a randomly generated baseline date for patients without TNF inhibitor use) and 6797 patients with missing data corresponding to the baseline date, 10 689 patients were included in the final analytical cohort (Figure 1).

To examine the association of incident use (vs nonuse) of TNF inhibitors with outcomes, we used an intention-to-treat–like approach, where patients with de novo TNF inhibitor use were considered part of the treated group until the end of follow-up, irrespective of subsequent treatment status. The study follow-up in treated patients began on the date of receiving the first de novo prescription of TNF inhibitors; while, to mitigate differential start of follow-up bias, untreated patients were enrolled on a randomly assigned date that was computer-generated based on the start dates in the treated group (modeling elapsed time from cohort entry to start of treatment).

### Exposure Variable

The primary exposure of interest was incident use of TNF inhibitors, defined as the de novo prescription of any TNF inhibitors (adalimumab, infliximab, certolizumab, and golimumab), preceded by the lack of any prescriptions of TNF inhibitors during the previous time period, while having a record of VA pharmacy enrollment during the same period. The administration of TNF inhibitors was modeled using an intention-to-treat–like approach. Patients who had no record of any TNF inhibitor prescription throughout the study period served as untreated comparators. We described actual exposure to anti-TNF therapy in the treated group by calculating the proportion of days covered by the drug during the follow-up period.^28,29^ Information about prescribed medications was obtained from the Decision Support System National Data Extracts outpatient and inpatient pharmacy files, as well as from Medicare Part D files for those eligible for such coverage.^30^ Medications obtained outside VA pharmacies were identified from non-VA medication files in the VA Corporate Data Warehouse.^31^

### Covariates

Data on patient demographics, socioeconomic characteristics, comorbidities, vital signs, medications, and laboratory characteristics corresponding to each patient’s baseline date were collected retrospectively through the VA Corporate Data Warehouse.^31^ We used self-reported race and ethnicity categories as reported in the VA Corporate Data Warehouse, including African American, Hispanic, White, and other, which included those who self-identified as Asian, American Indian, Pacific Islander, and other without further specification. Race and ethnicity data were included because they have known associations with kidney outcomes and are thus considered confounders. Information about comorbidities, vital signs, and laboratory values were retrospectively extracted from various data sources as described elsewhere.^26,32^ Baseline medication use was defined respectively as the presence of at least 1 prescription of at least 30 days’ supply, and vital and laboratory values were defined using the most proximate measurements during the 1 year preceding the baseline date. The median (IQR) time intervals between the laboratory measurement dates and baseline date were 85 (31-170) days for serum albumin, 82 (28-170) days for white blood cell count, and 275 (196-327) days for eGFR. eGFR was calculated using the Chronic Kidney Disease Epidemiology Collaboration (CKD-EPI) 2021 equation using serum creatinine and demographic data.^33^ None of the covariates were missing in the final analytical cohort.

### Outcome Assessment

Our co–primary outcomes were 30% decline in eGFR and all-cause mortality. Decline in eGFR in each patient was defined as at least 30% reduction in outpatient eGFR from baseline that was sustained for at least 2 consecutive measurements at least 30 days apart, unless the reduction was identified on the last available measurement during the follow-up period. There was a median (IQR) of 15 (7-32) outpatient eGFR measurements. All-cause mortality was ascertained by the VA Vital Status Files.^34^ Cardiovascular and infection-related mortality were identified from the National Death Index and treated as secondary outcomes of interest in a sensitivity analysis. Patients free of death were censored at the date of the last VA encounter, incident ESKD, or September 30, 2019, whichever occurred first, and death from causes other than cardiovascular or infection-related death were treated as a competing event in the sensitivity analysis.

### Statistical Analysis

Baseline patient characteristics were summarized for the entire analytical population and according to TNF inhibitor use status and presented as number and percentage for categorical variables and mean and SD for continuous variables with a normal distribution or median and IQR for those with a skewed distribution. The significance of differences in baseline patient characteristics in the overall cohort was established based on considerations of biologically or clinically meaningful differences, as even very small differences are rendered statistically significant by the large sample size. The associations of incident TNF inhibitor use with outcomes were assessed using the Kaplan-Meier method with log-rank tests and Cox proportional hazards models, with adjustment for potential confounders. Models were incrementally adjusted for the following baseline confounders based on theoretical considerations and their availability in this study: model 1 is adjusted for age, sex, and race; model 2 additionally adjusted for comorbidities (diabetes, myocardial infarction, peripheral vascular disease, ischemic heart disease, congestive heart failure, chronic pulmonary disease, liver disease, dementia, cancer, depression, and HIV/AIDS) and Charlson Comorbidity Index; model 3 additionally adjusted for body mass index (BMI; calculated as weight in kilograms divided by height in meters squared), systolic blood pressure, and eGFR; model 4 additionally included medications (proton pump inhibitors, renin-angiotensin-aldosterone system inhibitors, nonsteroidal anti-inflammatory drugs, immunomodulators [methotrexate, sulfasalazine, mesalamine, 6-mercaptopurine, and azathioprine], and corticosteroids); model 5 further included socioeconomic status (service connectedness and mean per-capita income); and model 6 further accounted for surrogate markers of inflammation (serum albumin and white blood cell count) and was treated as the main model. Variance inflation factors were calculated to examine substantial multicollinearity among these parameters, and values more than 5.0 were considered to indicate collinearity.

We performed several sensitivity analyses to evaluate the robustness of our main findings. The associations between TNF inhibitor use and outcomes were examined in subgroups divided by age, prevalent diabetes, BMI, eGFR, corticosteroid use, white blood cell count, and IBD diagnosis (ie, ulcerative colitis or Crohn disease) at baseline. Potential interactions were formally tested by including interaction terms. To further enrich our sample, we included 17 486 patients with missing covariates at baseline and repeated our main analysis after imputing missing data. Missing baseline covariates were imputed by multiple imputation (5 multiple imputation datasets created by chained equations). To account for potential confounding by medical indication (eg, age), the associations of TNF inhibitor use with outcomes were examined in a propensity score–matched cohort (Figure 1).^35,36^ Propensity scores for the likelihood of use vs nonuse of TNF inhibitors were calculated by logistic regression using all variables included in the main multivariable model (ie, model 6). We then matched treated patients with untreated comparators using a 1:1 nearest-neighbor matching without replacement, resulting in a total of 1930 patients in the propensity-matched cohort. Differences between variables in the propensity-matched cohort were examined by calculating standardized differences, and values less than 0.1 were considered acceptable for the matching. Additionally, the main analysis was repeated by excluding a variable of Charlson Comorbidity Index due to its potential collinearity with other variables (with an variance inflation factors of 5.60). Lastly, to examine the association of TNF inhibitor use with cause-specific mortality (ie, cardiovascular or infection-related death), subdistribution hazards models were performed by treating death from other causes as a competing event in the overall cohort (using the fully adjusted model) and in the propensity-matched cohort.^37^

Analyses were conducted using Stata MP version 17.1 (StataCorp) and SAS version 9.4 (SAS Institute). Analyses were conducted in December 2022 to February 2024.

## Results

### Patient Characteristics

Baseline patient characteristics overall and by TNF inhibitor use status in the overall cohort are summarized in Table 1. Among 10 689 patients (mean [SD] age, 67.4 [12.3] years; 9999 [93.5%] male) with incident IBD included in analysis, 1515 (14.2%) were newly initiated on TNF inhibitors. Overall, 1211 patients (11.3%) were African American, 437 patients (4.1%) were Hispanic, and 9016 patients (84.3%) were White; 3353 patients (31.4%) had diabetes. The mean (SD) BMI was 28.8 (5.8), and the mean (SD) eGFR was 77.2 (19.2) mL/min/1.73 m^2^. The most prescribed TNF inhibitor was adalimumab (793 patients [52.4%]), followed by infliximab (685 patients [45.2%]), certolizumab (34 patients [2.2%]), and golimumab (3 patients [0.2%]) (eTable 2 in Supplement 1). The mean (SD) proportion of days covered for anti-TNF therapy during the follow-up period in treated patients was 0.85 (0.26). Compared with patients without TNF inhibitor use, patients initiating anti-TNF therapy were younger and more likely to be service connected (Table 1). Patients initiating TNF inhibitors also had a lower prevalence of comorbidities, except liver disease and depression, and had higher white blood cell counts and eGFR levels and lower serum albumin levels. The use of proton pump inhibitors, nonsteroidal anti-inflammatory drugs, immunomodulators, and corticosteroids were more common in incident users (vs nonusers) of TNF inhibitors (Table 1). In the propensity-matched cohort, baseline characteristics were well balanced between new users and nonusers of TNF inhibitors (eTable 3 in Supplement 1).

**Table 1.** Baseline Patient Characteristics by TNF Inhibitor Use Status in the Overall Cohort

Characteristic	Patients, No. (%)		
	Total (N = 10 689)	No TNF inhibitor use (n = 9174)	TNF inhibitor use (n = 1515)
Age, mean (SD), y	67.4 (12.3)	68.7 (11.5)	59.4 (13.5)
Sex			
Male	9999 (93.5)	8605 (93.8)	1394 (92.0)
Female	690 (6.5)	569 (6.2)	121 (8.0)
Race and ethnicity			
African American	1211 (11.3)	1016 (11.1)	195 (12.9)
Hispanic	437 (4.1)	364 (83.3)	73 (16.7)
White	9016 (84.3)	7771 (84.7)	1245 (82.2)
Others^a^	462 (4.3)	387 (4.2)	75 (5.0)
Marital status			
Single	878 (8.2)	690 (7.5)	188 (12.4)
Married	6307 (59.0)	5494 (59.9)	813 (53.7)
Divorced	2489 (23.3)	2053 (22.4)	436 (28.8)
Widowed	885 (8.3)	819 (8.9)	66 (4.4)
Unknown	130 (1.2)	118 (1.3)	12 (0.8)
Service connected	6119 (57.2)	5102 (55.6)	1017 (67.1)
Median per capita income (IQR), $	17 392 (4400-34 600)	17 343 (3012-34 800)	17 532 (8652-34 116)
BMI	28.8 (5.8)	29.0 (5.7)	28.1 (5.9)
Systolic blood pressure, mm Hg	129.4 (16.9)	129.7 (16.8)	128.0 (17.6)
IBD diagnosis^b^			
Ulcerative colitis	6444 (60.3)	5842 (63.7)	602 (39.7)
Crohn disease	4237 (39.6)	3324 (36.2)	913 (60.3)
Comorbidities			
Diabetes	3353 (31.4)	2958 (32.2)	395 (26.1)
Myocardial infarction	1211 (11.3)	1082 (11.8)	129 (8.5)
Peripheral vascular disease	1814 (17.0)	1649 (18.0)	165 (10.9)
Ischemic heart disease	3445 (32.2)	3063 (33.4)	382 (25.2)
Congestive heart failure	1409 (13.2)	1267 (13.8)	142 (9.4)
Chronic lung disease	3295 (30.8)	2835 (30.9)	460 (30.4)
Liver disease	975 (9.1)	812 (8.9)	163 (10.8)
Dementia	465 (4.4)	430 (4.7)	35 (2.3)
Malignant neoplasm	1983 (18.6)	1796 (19.6)	187 (12.3)
Depression	1872 (17.5)	1505 (16.4)	367 (24.2)
HIV/AIDS	65 (0.6)	60 (0.7)	5 (0.3)
Charlson Comorbidity Index, median (IQR)	2 (0-4)	2 (0-4)	2 (0-3)
Medications			
Statins	4771 (44.6)	4208 (45.9)	563 (37.2)
Proton pump inhibitors	4153 (38.9)	3327 (36.3)	826 (54.5)
RAAS inhibitors	3710 (34.7)	3245 (35.4)	465 (30.7)
Calcium channel blockers	2149 (20.1)	1865 (20.3)	284 (18.7)
Thiazide diuretics	1997 (18.7)	1739 (19.0)	258 (17.0)
Loop diuretics	1006 (9.4)	875 (9.5)	131 (8.6)
Potassium-sparing diuretics	370 (3.5)	330 (3.6)	40 (2.6)
NSAIDs	3053 (28.6)	2602 (28.4)	451 (29.8)
Methotrexate	94 (0.9)	45 (0.5)	49 (3.2)
Sulfasalazine	515 (4.8)	454 (4.9)	61 (4.0)
Mesalamine	3356 (31.4)	2700 (29.4)	656 (43.3)
6-Mercaptopurine	632 (5.9)	346 (3.8)	286 (18.9)
Azathioprine	608 (5.7)	228 (2.5)	380 (25.1)
Corticosteroids	1310 (12.3)	478 (5.2)	832 (54.9)
Laboratory findings, mean (SD)			
Serum albumin, g/dL	3.9 (0.5)	4.0 (0.5)	3.6 (0.7)
White blood cell count, cells/μL	7400 (2900)	7200 (2800)	8300 (3300)
eGFR, mL/min/1.73 m^2^	77.2 (19.2)	76.0 (18.9)	84.2 (20.0)
eGFR <60 mL/min/1.73 m^2^, No. (%)	1957 (18.3)	1773 (19.3)	184 (12.2)

Abbreviations: eGFR, estimated glomerular filtration rate; IBD, inflammatory bowel disease; NSAIDs, non-steroidal anti-inflammatory drugs; RAAS, renin-angiotensin-aldosterone system; TNF, tumor necrosis factor.

SI conversion factors: To convert albumin to grams per liter, multiply by 10; white blood cells to ×10^9^/L, multiply by 0.001.

^a^ Includes individuals who identified as Asian, American Indian, or Pacific Islander and those who identified as other race or ethnicity without providing further information.

^b^ Eight patients had both ulcerative colitis and Crohn disease diagnoses.

### Association of Incident TNF Inhibitor Use With Decline in eGFR

**Figure 2.**
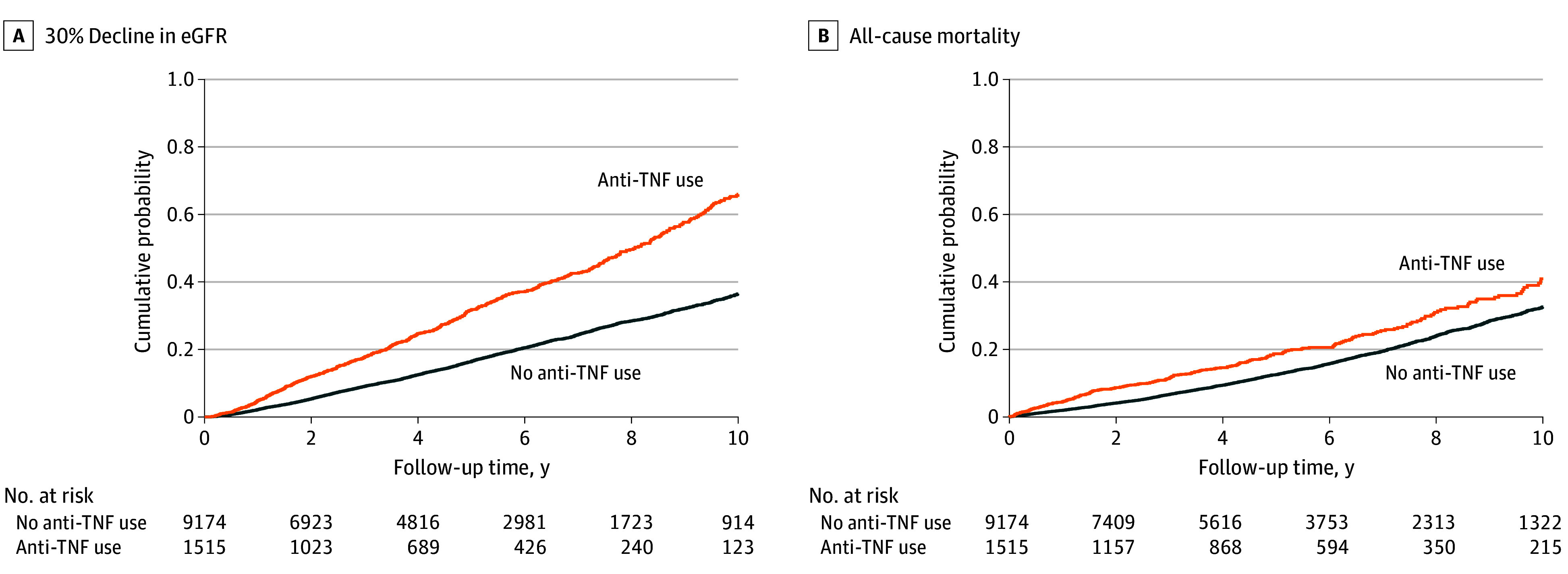
Kaplan-Meier Cumulative Event Curves for at Least 30% Decline in Estimated Glomerular Filtration Rate (eGFR) and All-Cause Mortality Associated With Incident Use (vs Nonuse) of Tumor Necrosis Factor (TNF) Inhibitors in the Overall Cohort Log-rank *P* < .001 for both. Kaplan-Meier curves were demographically adjusted for age, sex, and race.

Among 10 689 patients in the overall cohort, 3367 patients (crude rate, 66.1 [95% CI, 63.9-68.4] per 1000 patient-years [PYs]), including 607 users (crude rate, 92.7 [95% CI, 85.6-100.4] per 1000 PYs) and 2760 nonusers (crude rate, 62.2 [95% CI, 59.9-64.6] per 1000 PYs) of TNF inhibitors, experienced at least 30% decline in eGFR over a median (IQR) follow-up of 4.1 (1.9-7.0) years (new users, 3.5 [1.5-6.4] years; nonusers, 4.2 [2.0-7.0] years). As shown in Figure 2A, compared with patients without anti-TNF therapy, those who newly initiated TNF inhibitors had a higher cumulative incidence of 30% decline in eGFR (log-rank *P* < .001).

Table 2 shows the association of the use (vs nonuse) of TNF inhibitors with decline in eGFR across multivariable-adjusted models. In demographically adjusted model (ie, model 1), the use (vs nonuse) of TNF inhibitors was significantly associated with higher risk of decline in eGFR (adjusted hazard ratio [HR], 1.71 [95% CI, 1.55-1.87]). Although this association was modified after further multivariable adjustments, the higher risk of 30% decline in eGFR associated with the use (vs nonuse) of TNF inhibitors remained significant in the fully adjusted model (model 6: adjusted HR, 1.34 [95% CI, 1.18-1.52]).

**Table 2.** Association of Incident Tumor Necrosis Factor Inhibitor Use (vs Nonuse) With at Least 30% Decline in eGFR and All-Cause Mortality in the Overall Cohort

Model^a^	HR (95% CI)
**≥30% Reduction in eGFR**	
1	1.71 (1.55-1.87)
2	1.81 (1.65-1.99)
3	1.86 (1.69-2.04)
4	1.60 (1.42-1.81)
5	1.58 (1.40-1.78)
6	1.34 (1.18-1.52)
**All-cause mortality**	
1	1.44 (1.26-1.65)
2	1.56 (1.36-1.78)
3	1.50 (1.31-1.72)
4	1.31 (1.11-1.54)
5	1.31 (1.11-1.55)
6	1.02 (0.86-1.21)

Abbreviation: eGFR, estimated glomerular filtration rate.

^a^ Model 1 is adjusted for age, sex, and race; model 2 is adjusted for the variables in model 1 plus comorbidities (diabetes, myocardial infarction, peripheral vascular disease, ischemic heart disease, congestive heart failure, chronic pulmonary disease, liver disease, dementia, cancer, depression, and HIV/AIDS), and Charlson Comorbidity Index; model 3 is adjusted for the variables in model 2 plus body mass index, systolic blood pressure, and eGFR; model 4 is adjusted for the variables in model 3 plus medications (proton pump inhibitors, renin-angiotensin-aldosterone system inhibitors, nonsteroidal anti-inflammatory drugs, methotrexate, sulfasalazine, mesalamine, 6-mercaptopurine, azathioprine, and corticosteroids); model 5 is adjusted for the variables in model 4 plus socioeconomic status (service connectedness and mean per capita income); and model 6 is adjusted for the variables in model 5 plus surrogate markers of inflammation (serum albumin and white blood cell count).

**Figure 3.**
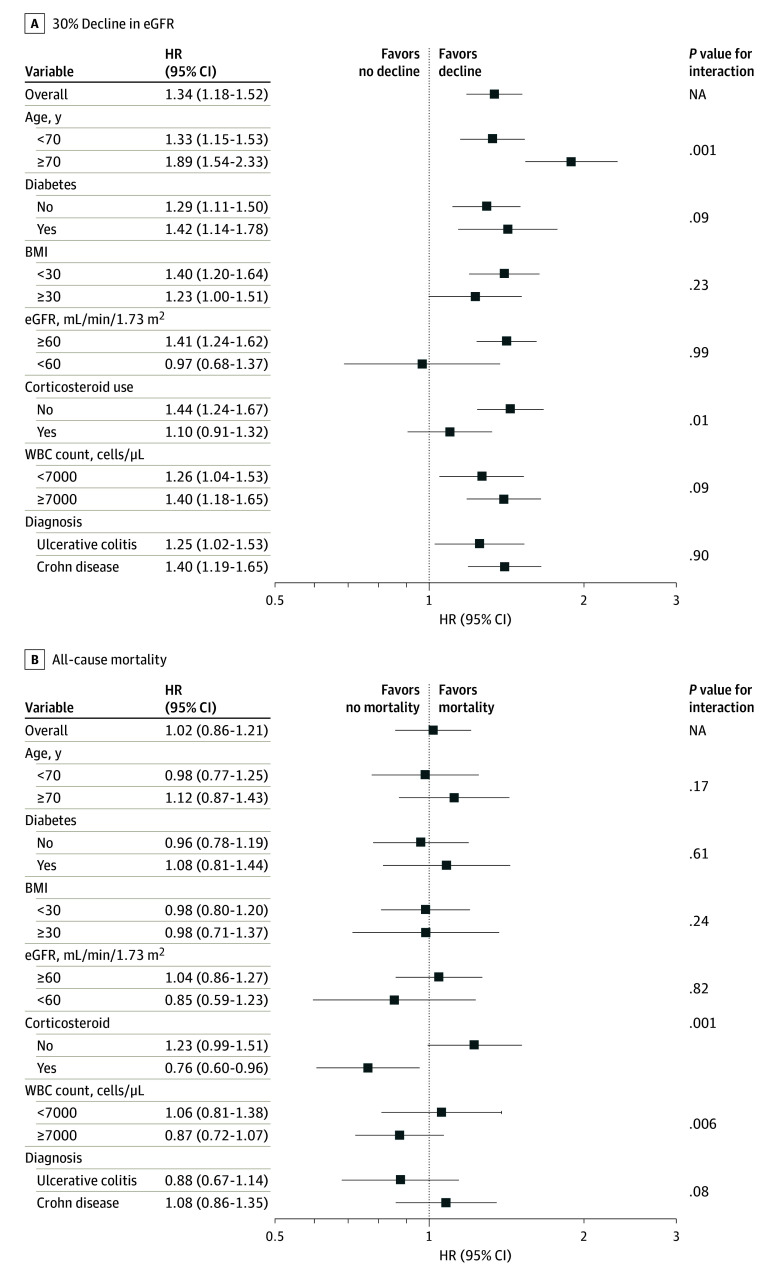
Association of Incident Tumor Necrosis Factor (TNF) Inhibitor Use (vs Nonuse) With at Least 30% Decline in Estimated Glomerular Filtration Rate (eGFR) and All-Cause Mortality in Selected Subgroups in the Overall Cohort Data are adjusted for variables included in model 6 (ie, fully adjusted model). BMI, indicates body mass index (calculated as weight in kilograms divided by height in meters squared); HR, hazard ratio; WBC, white blood cell count (to convert to ×10^9^/L, multiply by 0.001). SI conversion factors: To convert albumin to grams per liter, multiply by 10; white blood cells to ×10^9^/L, multiply by 0.001.

Findings were similarly observed in selected subgroups (Figure 3A) and were robust to sensitivity analyses accounting for missing data, confounding by indication in the propensity-matched cohort, and potential multicollinearity (eFigure and eTables 4-6 in Supplement 1). With a few exceptions, the association of TNF inhibitor use to the risk of decline in eGFR was more evident in subgroups of patients aged 70 years and older and those without corticosteroid use than in their counterparts, with statistically significant interactions (Figure 3A).

### Association of Incident TNF Inhibitor Use With All-Cause Mortality

During a median (IQR) follow-up of 5.0 (2.5-8.0) years (new users, 4.8 [2.2-7.9] years; nonusers, 5.0 [2.6-8.0] years), a total of 2502 all-cause deaths occurred (crude rate, 42.3 [95% CI, 40.3-44.0] per 1000 PYs), including 262 (crude rate, 32.6 [95% CI, 28.9-36.8] per 1000 PYs) deaths in patients with TNF inhibitor use and 2240 (crude rate, 43.8 [95% CI, 42.1-45.7] per 1000 PYs) deaths in patients without TNF inhibitor use. As depicted in Figure 2B, patients with (vs without) TNF inhibitor use had a higher cumulative incidence of all-cause mortality (log-rank *P* < .001). Table 2 shows multivariable adjusted HRs of all-cause mortality associated with incident use of TNF inhibitors. The significant association of TNF inhibitor use with higher all-cause mortality was attenuated after incremental multivariable adjustment and was no longer present in the fully adjusted model (model 6 adjusted HR, 1.02 [95% CI, 0.86-1.21]).

Results were similar in various sensitivity analyses (Figure 3B; eFigure and eTables 4-6 in Supplement 1). With 1 exception, the use of TNF inhibitors was significantly associated with lower mortality in a subgroup of patients with corticosteroid use, with a statistically significant interaction. There was no significant association of incident TNF inhibitor use with cardiovascular or infection-related mortality (eTable 7 in Supplement 1).

## Discussion

In this large nationwide cohort study of US veterans with incident IBD, we examined the association of incident TNF inhibitor use with at least 30% decline in eGFR and all-cause mortality and found that incident use (vs non-use) of TNF inhibitors was significantly associated with higher subsequent risk of decline in eGFR but was not associated with risk of all-cause mortality. Findings were generally consistent in several subgroups, after accounting for missing data, and using a propensity score–matching approach.

Although our observational study cannot conclude any causal relationships, there are a few plausible explanations for the observed association between anti-TNF therapy and progressive kidney function decline. Many previous studies, including clinical trials and postmarketing surveillance studies, have documented autoimmune-mediated phenomena as 1 of the common biologically related adverse events in IBD,^6,38^ among which anti-TNF–induced lupus is a well-recognized clinical entity that affects various organs, including the kidneys.^39,40^ According to a 2022 systematic review and meta-analysis evaluating the incidence of confirmed anti-TNF–induced lupus in patients with IBD, the incident rate of kidney involvement was 8.7%.^40^ Along with the findings reported by Dai et al, several case reports and case series of patients with IBD have reported patients developed glomerulonephritis and tubulointerstitial nephritis following the initiation of biologic therapy, leading to progressive eGFR decline.^41,42,43^ Our finding of no significant association of anti-TNF therapy with the risk of decline in eGFR among patients using corticosteroids may support this explanation (eg, by suppressing anti-TNF-induced autoimmune responses). Meanwhile, an eGFR decline could also be observed due to an increase in serum creatinine generation as a consequence of gaining muscle mass.^44,45,46^ Albeit speculative, it is therefore possible that the gain of muscle mass associated with improvement of IBD symptoms following anti-TNF therapy and resultant amelioration of nutritional deficiency led to an apparent decline in eGFR among patients with incident TNF inhibitor use. Most importantly, in line with a 2022 expert consensus about kidney function monitoring in IBD,^17^ our findings on the association of TNF inhibitor use with progressive eGFR decline suggest the need for careful assessment and monitoring of kidney function when initiating anti-TNF therapy in patients with IBD.

Regarding the association of incident TNF inhibitor use with all-cause mortality in patients with IBD, a few observational studies have reported a lower risk of mortality associated with incident TNF inhibitor use.^22,23^ However, these previous studies have assessed the mortality risk associated with new anti-TNF therapy compared with prolonged use of corticosteroids; hence, it remained unclear whether patients with new anti-TNF drug use could have a survival benefit compared with nonusers of such therapy, irrespective of prolonged use of corticosteroids. While longstanding corticosteroid use among patients with IBD remains common, the treatment of IBD without corticosteroid use toward corticosteroid-free remission is also a possible scenario in the management of IBD in clinical settings.^47^ In this study, we compared the mortality risk between de novo and never users of TNF inhibitors, irrespective of concomitant drugs, and found no significant survival benefit associated with de novo anti-TNF therapy (vs nonuse) among patients with incident IBD, which supports findings from a few prior studies demonstrating no significant association between anti-TNF therapy and mortality.^10,24,25^

Notably, in the subgroup analysis, we observed a significantly lower risk of all-cause mortality associated with anti-TNF therapy in patients with corticosteroid use. The precise mechanisms underlying this observation remain unclear. Given the well-known corticosteroid-sparing effects of biologics in IBD,^47,48^ the observed survival benefit might be owing at least in part to corticosteroid-sparing effects following the administration of biologic therapy.

### Limitations

Despite several advantages of this study, including the rigorous incident new user design to define TNF inhibitor exposure among patients with incident IBD, our results must be interpreted in light of some limitations. Our cohort consisted of predominantly (93.5%) male US veterans, and hence the results may not be generalizable to female or nonveteran populations. The exposure of interest was the use of TNF inhibitors, hence the conclusions may not apply to biologics in other classes. Although we assessed at least 30% decline in eGFR as a valid surrogate end point for CKD progression,^49,50^ due to the low number of incident kidney failure requiring kidney replacement in this cohort, we were unable to examine the association of incident TNF inhibitor use with a tangible kidney outcome. Due to the small number of patients with serum cystatin-C measurements available, we were unable to assess cystatin-C–based eGFR. As with all observational studies, we cannot eliminate the possibility of unmeasured (eg, IBD severity and proteinuria) and residual (eg, doses of immunomodulators) confounders that might have affected the association between incident TNF inhibitor use and outcomes. It is also possible that characteristics of patients eligible for anti-TNF therapy differed from those not eligible for anti-TNF therapy. This possibility cannot be fully excluded despite the effort to minimize confounding by indication and differential start of follow-up. Nevertheless, given the scarcity of clinical trial evidence on the effect of TNF inhibitors on decline in eGFR and all-cause mortality, the findings from this designed observational study may offer meaningful evidence currently available in the field.

## Conclusions

In this large nationwide cohort study of 10 689 US veterans with incident IBD, we found that incident use of TNF inhibitors was independently associated with higher risk of progressive kidney function decline but was not associated with risk of all-cause mortality. Further studies are needed to confirm findings and to examine potentially distinct pathophysiologic contributions of incident TNF inhibitor use to kidney and nonkidney outcomes in patients with IBD.

## Notes

Supplement 1. **eTable 1.** *ICD-9* and *ICD-10* Codes Used to Define Inflammatory Bowel Disease **eTable 2.** Prescribed TNF Inhibitors in the Overall Cohort **eTable 3.** Baseline Patient Characteristics According to TNF Inhibitor Use Status in the Propensity-Matched Cohort **eTable 4.** Association of Incident TNF Inhibitor Use (vs Nonuse) With a 30% Decline in eGFR and All-Cause Mortality in the Overall Cohort With Multiple Imputations for Missing Data **eTable 5.** Association of Incident TNF Inhibitor Use (vs Nonuse) With a 30% Decline in eGFR and All-Cause Mortality in the Propensity-Matched Cohort **eTable 6.** Association of Incident TNF Inhibitor Use (vs Nonuse) With a 30% Decline in eGFR and All-Cause Mortality in the Overall Cohort After Excluding a Variable of Charlson Comorbidity Index From the Multivariable Adjustment **eTable 7.** Association of Incident TNF Inhibitor Use (vs Nonuse) With Cardiovascular and Infection-Related Mortality Using Competing Risk Regression Models by Treating Other Causes of Death as a Competing Event in the Overall and Propensity-Matched Cohort **eFigure.** Kaplan-Meier Cumulative Event Curves for a 30% Decline in eGFR and All-Cause Mortality Associated With Incident Use (vs Nonuse) of TNF Inhibitors in the Propensity-Matched Cohort

Supplement 2.
Data Sharing Statement
