# Supplementary material for: Anti–Tumor Necrosis Factor Therapy and Risk of Kidney Function Decline and Mortality in Inflammatory Bowel Disease
Source: JAMA Netw Open. 2024 Apr 16;7(4):e246822. doi: 10.1001/jamanetworkopen.2024.6822 (PMC11022116; doi:10.1001/jamanetworkopen.2024.6822)
Supplement: Supplement 1. — eTable 1. ICD-9 and ICD-10 Codes Used to Define Inflammatory Bowel Disease eTable 2. Prescribed TNF Inhibitors in the Overall Cohort eTable 3. Baseline Patient Characteristics According to TNF Inhibitor Use Status in the Propensity-Matched Cohort eTable 4. Association of Incident TNF Inhibitor Use (vs Nonuse) With a 30% Decline in eGFR and All-Cause Mortality in the Overall Cohort With Multiple Imputations for Missing Data eTable 5. Association of Incident TNF Inhibitor Use (vs Nonuse) With a 30% Decline in eGFR and All-Cause Mortality in the Propensity-Matched Cohort eTable 6. Association of Incident TNF Inhibitor Use (vs Nonuse) With a 30% Decline in eGFR and All-Cause Mortality in the Overall Cohort After Excluding a Variable of Charlson Comorbidity Index From the Multivariable Adjustment eTable 7. Association of Incident TNF Inhibitor Use (vs Nonuse) With Cardiovascular and Infection-Related Mortality Using Competing Risk Regression Models by Treating Other Causes of Death as a Competing Event in the Overall and Propensity-Matched Cohort eFigure. Kaplan-Meier Cumulative Event Curves for a 30% Decline in eGFR and All-Cause Mortality Associated With Incident Use (vs Nonuse) of TNF Inhibitors in the Propensity-Matched Cohort [file jamanetwopen-e246822-s001.pdf]

Supplemental Online Content

Sumida K, Shrestha P, Mallisetty Y, et al. Anti–tumor necrosis factor therapy and risk of kidney function decline and mortality in inflammatory bowel disease. *JAMA Netw Open*. 2024;7(4):e246822. doi:10.1001/jamanetworkopen.2024.6822

eTable 1. *ICD-9* and *ICD-10* Codes Used to Define Inflammatory Bowel Disease

eTable 2. Prescribed TNF Inhibitors in the Overall Cohort

eTable 3. Baseline Patient Characteristics According to TNF Inhibitor Use Status in the Propensity-Matched Cohort

eTable 4. Association of Incident TNF Inhibitor Use (vs Nonuse) With a 30% Decline in eGFR and All-Cause Mortality in the Overall Cohort With Multiple Imputations for Missing Data

eTable 5. Association of Incident TNF Inhibitor Use (vs Nonuse) With a 30% Decline in eGFR and All-Cause Mortality in the Propensity-Matched Cohort

eTable 6. Association of Incident TNF Inhibitor Use (vs Nonuse) With a 30% Decline in eGFR and All-Cause Mortality in the Overall Cohort After Excluding a Variable of Charlson Comorbidity Index From the Multivariable Adjustment

eTable 7. Association of Incident TNF Inhibitor Use (vs Nonuse) With Cardiovascular and Infection-Related Mortality Using Competing Risk Regression Models by Treating Other Causes of Death as a Competing Event in the Overall and Propensity-Matched Cohort

eFigure. Kaplan-Meier Cumulative Event Curves for a 30% Decline in eGFR and All-Cause Mortality Associated With Incident Use (vs Nonuse) of TNF Inhibitors in the Propensity-Matched Cohort

This supplemental material has been provided by the authors to give readers additional information about their work.

© 2024 Sumida K et al. *JAMA Network Open.*

eTable 1. ICD-9/10 codes used to define inflammatory bowel disease

	ICD-9	ICD-10
Crohn’s disease	555.0, 555.1, 555.2, 555.9	K50.0, K50.1, K50.8, K50.9
Ulcerative colitis	556.0, 556.1, 556.2, 556.3, 556.4, 556.5, 556.6, 556.8, 556.9	K51.0, K51.2, K51.3, K51.4, K51.5, K51.8, K51.9

Abbreviations: ICD = International Classification of Diseases

© 2024 Sumida K et al. *JAMA Network Open.*

eTable 2. Name and number (%) of prescribed TNF-alpha inhibitors in the overall cohort (n=10,689)

Name	Number (%)
Adalimumab	793 (52.4)
Infliximab	685 (45.2)
Certolizumab	34 (2.2)
Golimumab	3 (0.2)

Note: Data are based on the drug names available in the VA medication files.

Abbreviations: TNF = tumor necrosis factor

© 2024 Sumida K et al. *JAMA Network Open.*

eTable 3. Baseline patient characteristics according to TNF-alpha inhibitor use status in the propensity-matched cohort (n=1,930)

	TNF-alpha inhibitors use		
	**No**	**Yes**	**Std. Diff.**
Number of patients	965	965	
Age, mean (SD), year	61.4±12.3	62.2±12.4	-0.07
Sex, n (%)			0.0001
Men	883 (91.5)	883 (91.5)	
Women	82 (8.5)	82 (8.5)	
Race, n (%)			0.03
White	774 (80.2)	796 (82.5)	
African American	149 (15.4)	120 (12.4)	
Others[a]	42 (4.4)	49 (5.1)	
Service connected, n (%)	656 (68.0)	631 (65.4)	0.05
Median per capita income (IQI), $	18,413 (7,200-33,876)	18,276 (9,036-34,736)	-0.07
Body mass index (kg/m^2)	28.5±6.0	28.5±6.0	-0.001
Systolic blood pressure (mmHg)	129.0±17.9	128.7±17.7	0.02
Comorbidities, n (%)			
Diabetes mellitus	262 (27.2)	273 (28.3)	-0.03
Myocardial infarction	86 (8.9)	93 (9.6)	-0.03
Peripheral vascular disease	118 (12.2)	134 (13.9)	-0.05
Ischemic heart disease	241 (25.0)	279 (28.9)	-0.09
Congestive heart failure	92 (9.5)	107 (11.1)	-0.05
Chronic lung disease	331 (34.3)	325 (33.7)	0.01
Liver disease	113 (11.7)	120 (12.4)	-0.02
Dementia	25 (2.6)	29 (3.0)	-0.03
Malignancies	128 (13.3)	134 (13.9)	-0.02
Depression	239 (24.8)	235 (24.4)	0.01
HIV/AIDS	6 (0.6)	4 (0.4)	0.0001
Charlson Comorbidity Index	2 (0-4)	2 (1-4)	0.03
Medications, n (%)			
Proton pump inhibitors	496 (51.4)	508 (52.6)	-0.02
RAAS inhibitors	316 (32.7)	318 (33.0)	-0.01
NSAIDs	304 (31.5)	297 (30.8)	0.02

© 2024 Sumida K et al. *JAMA Network Open.*

Methotrexate	26 (2.7)	26 (2.7)	0.0001
Sulfasalazine	48 (5.0)	48 (5.0)	0.0001
Mesalamine	439 (45.5)	417 (43.2)	0.05
6-mercaptopurine	160 (16.6)	132 (13.7)	0.08
Azathioprine	134 (13.9)	125 (13.0)	0.03
Corticosteroids	307 (31.8)	321 (33.3)	-0.03
Laboratory parameters			
Serum albumin (g/dL)	3.7±0.6	3.7±0.6	-0.06
White blood cell count (1,000/uL)	8.1±4.7	8.0±2.9	0.03
Estimated GFR (mL/min/1.73 m^2)	82.8±20.5	81.6±19.5	0.06

Note: Data are presented as number (percentage), mean±SD, or median (interquartile interval).

[a]Includes individuals who identified as Asian, American Indian, or Pacific Islander and those who identified as other race or ethnicity without providing further information.

Abbreviations: GFR = glomerular filtration rate; HIV/AIDS = human immunodeficiency virus/acquired immunodeficiency syndrome; IQI = interquartile interval; NSAIDs = non-steroidal anti-inflammatory drugs; RAAS = renin-angiotensin-aldosterone system; SD = standard deviation; TNF = tumor necrosis factor

© 2024 Sumida K et al. *JAMA Network Open.*

eTable 4. Association of incident TNF-alpha inhibitor use (vs. non-use) with (A) a 30% decline in eGFR and (B) all-cause mortality in the overall cohort with multiple imputations for missing data (n=17,486)

(A) A 30% decline in eGFR

Adjusted hazard ratio* (95% CI)	1.65 (1.47, 1.85)

(B) All-cause mortality

Adjusted hazard ratio* (95% CI)	0.95 (0.82, 1.09)

*Data were adjusted for variables included in model 6 (i.e., fully adjusted model).

Abbreviations: CI = confidence interval; eGFR = estimated glomerular filtration rate; TNF = tumor necrosis factor

© 2024 Sumida K et al. *JAMA Network Open.*

eTable 5. Association of incident TNF-alpha inhibitor use (vs. non-use) with (A) a 30% decline in eGFR and (B) all-cause mortality in the propensity-matched cohort (n=1,930)

(A) A 30% decline in eGFR

No. of patients	Events	Incidence rate (95% CI) per 1,000 person-years	Hazard ratio (95% CI)
1,930	700	83.2 (77.3, 89.6)	1.26 (1.09, 1.47)

(B) All-cause mortality

No. of patients	Events	Incidence rate (95% CI) per 1,000 person-years	Hazard ratio (95% CI)
1,930	426	42.3 (38.5, 46.5)	0.91 (0.75, 1.11)

Abbreviations: CI = confidence interval; eGFR = estimated glomerular filtration rate; TNF = tumor necrosis factor

© 2024 Sumida K et al. *JAMA Network Open.*

eTable 6. Association of incident TNF-alpha inhibitor use (vs. non-use) with (A) a 30% decline in eGFR and (B) all-cause mortality in the overall cohort after excluding a variable of Charlson Comorbidity Index from the multivariable adjustment (n=10,689)

(A) A 30% decline in eGFR

Adjusted hazard ratio* (95% CI)	1.34 (1.18, 1.52)

(B) All-cause mortality

Adjusted hazard ratio* (95% CI)	0.87 (0.69, 1.11)

*Data were adjusted for variables included in model 6 (i.e., fully adjusted model) minus Charlson Comorbidity Index.

Abbreviations: CI = confidence interval; eGFR = estimated glomerular filtration rate; TNF = tumor necrosis factor

© 2024 Sumida K et al. *JAMA Network Open.*

eTable 7. Association of incident TNF-alpha inhibitor use (vs. non-use) with (A) cardiovascular and (B) infection-related mortality using competing risk regression models by treating other causes of death as a competing event in the (a) overall (n=10,689) and (b) propensity-matched cohort (n=1,930)

(A) Cardiovascular mortality

(a) Overall cohort

No. of patients	Primary events (*n*)	Competing events (*n*)	Adjusted sub-hazard ratio* (95% CI)
10,689	525	1,977	1.20 (0.82, 1.76)

(b) Propensity-matched cohort

No. of patients	Primary events (*n*)	Competing events (*n*)	Sub-hazard ratio (95% CI)
1,930	82	344	1.11 (0.72, 1.72)

(B) Infection-related mortality

(a) Overall cohort

No. of patients	Primary events (*n*)	Competing events (*n*)	Adjusted sub-hazard ratio* (95% CI)
10,689	84	2,418	1.30 (0.54, 2.96)

(b) Propensity-matched cohort

No. of patients	Primary events (*n*)	Competing events (*n*)	Sub-hazard ratio (95% CI)
1,930	17	409	0.78 (0.30, 2.07)

*Data were adjusted for variables included in model 6 (i.e., fully adjusted model).

Abbreviations: CI = confidence interval**;** TNF = tumor necrosis factor

© 2024 Sumida K et al. *JAMA Network Open.*

eFigure. Kaplan-Meier cumulative event curves for (A) a 30% decline in eGFR and (B) all-cause mortality associated with incident use (vs. non-use) of TNF-alpha inhibitors in the propensity-matched cohort (n=1,930)

(A) A 30% decline in eGFR

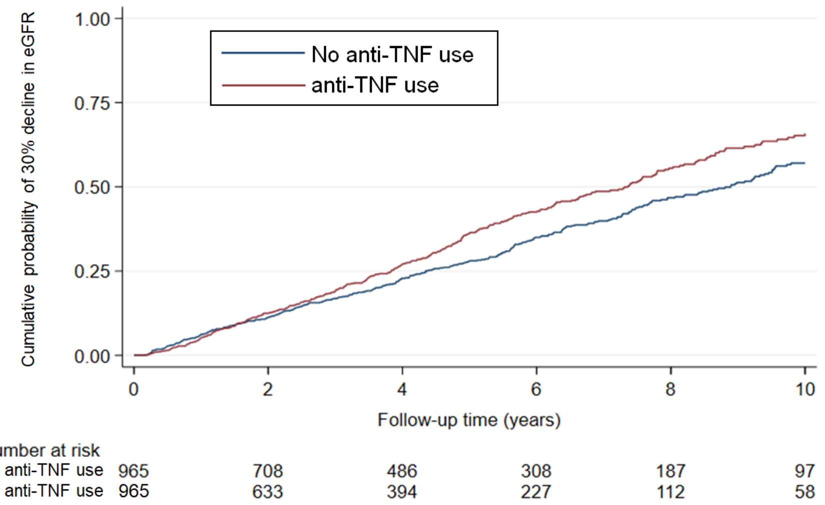

(B) All-cause mortality

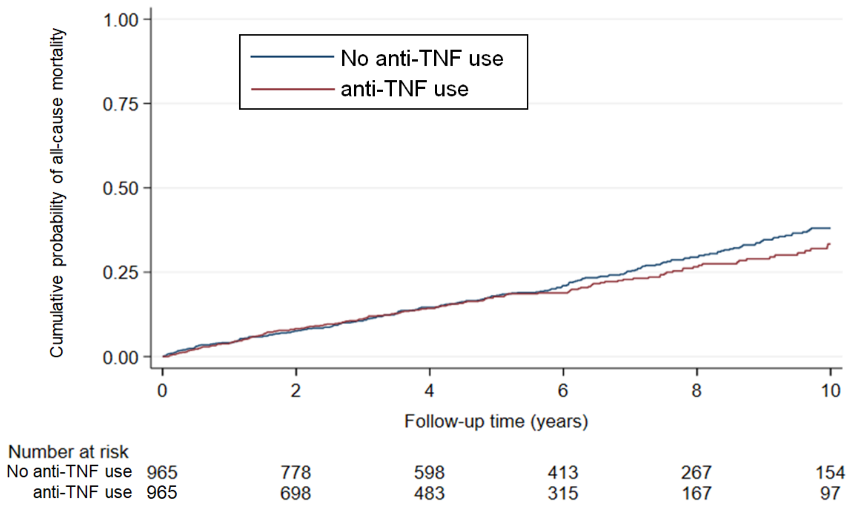

Note: Log-rank *P* <.01 (A) and *P*=0.35 (B).

Abbreviations: eGFR = estimated glomerular filtration rate; TNF = tumor necrosis factor

© 2024 Sumida K et al. *JAMA Network Open.*
