# Supplementary material for: Anti–Tumor Necrosis Factor Therapy and Risk of Kidney Function Decline and Mortality in Inflammatory Bowel Disease
Source: JAMA Netw Open. 2024 Apr 16;7(4):e246822. doi: 10.1001/jamanetworkopen.2024.6822 (PMC11022116; doi:10.1001/jamanetworkopen.2024.6822)
Supplement: Supplement 2. — Data Sharing Statement [file jamanetwopen-e246822-s002.pdf]

Data Sharing Statement

Sumida. Anti–Tumor Necrosis Factor Therapy and Risk of Kidney Function Decline and Mortality in Inflammatory Bowel Disease. *JAMA Netw Open*. Published April 16, 2024. doi:10.1001/jamanetworkopen.2024.6822

Data
Data available: No

Additional Information
Explanation for why data not available: VA regulations preclude the sharing of individual patient data with outside entities. De-identified data may be shared based on individual requests provided proper approvals are obtained.
